# IKEA effect and empathy for robots: Can assembly strengthen human-agent relationships?

**DOI:** 10.1371/journal.pone.0327524

**Published:** 2025-07-09

**Authors:** Takahiro Tsumura, Seiji Yamada

**Affiliations:** 1 Faculty of Information Networking for Innovation and Design, Toyo University, Tokyo, Japan; 2 National Institute of Informatics, Tokyo, Japan; 3 The Graduate University for Advanced Studies, SOKENDAI, Kanagawa, Japan; University of Pavia: Universita degli Studi di Pavia, ITALY

## Abstract

Cooperative relationships between humans and agents are becoming more important for the social coexistence of anthropomorphic agents, including virtual agents and robots. One way to improve the relationship between humans and agents is for humans to empathize with agents. Empathy can increase human acceptance. In this study, we focus on the IKEA effect in creating agents and examine empathy through interpersonal relationships. We conducted a robot assembly task in which participants either cooperatively built the same robot or individually assembled their own. The results showed that the IKEA effect promoted empathy toward the agent regardless of the relationship between participants. However, participants did not show a significant change in empathy levels from before to after the task. These results suggest that regardless of the relationship between participants, the IKEA effect can promote empathy toward the agent.

## Introduction

Intelligent artifacts such as ChatGPT, generative AI, and social robots (hereafter referred to as agents) are increasingly integrated into human society. Humans live in social environments and interact with various tools, sometimes treating agents as if they were human. According to the media equation theory, humans tend to interact with artificial agents as if they were interacting with real people [1]. Nevertheless, the growing familiarity with AI-based agents like ChatGPT and Siri has revealed that people do not always attribute human-like qualities to them. As the presence of agents in daily life grows, understanding how humans perceive and interact with them—not only as tools but also as potential social counterparts—has become an increasingly important research concern.

A fundamental challenge in human-agent interaction is fostering acceptance and trust. Previous research on AI ethics and trustworthiness has primarily focused on ensuring that AI systems function in a reliable and fair manner. Ryan [2] argued that even highly advanced AI systems should not inherently be considered trustworthy; rather, trust should be placed in the organizations and individuals managing these technologies. Similarly, Belk [3] examined the role of AI in service industries, highlighting its implications for public policy and technological applications. AI ethics has also been explored from an applied ethics perspective, as discussed by Hallamaa and Kalliokoski [4].

Beyond trust, empathy has been recognized as a key factor in shaping human-agent relationships. While trust is foundational, empathy serves as an emotional bridge that enables deeper acceptance of agents in human-centric environments. By cultivating empathy, users may not only trust agents’ decisions but also feel more emotionally connected to them. Wirtz and Pitardi [5] emphasized that while AI-driven services can enhance customer experience and efficiency, they also raise ethical, fairness, and privacy concerns. In medical AI applications, Kumar *et al*. [6] noted that public trust and ethical considerations remain central to discussions on AI integration. Moreover, research by Maehigashi *et al*. [7] demonstrated that even subtle behavioral cues from AI systems—such as timing in robotic responses—can significantly affect trust dynamics.

Just as trust is essential in human-agent interaction, humans often develop emotional responses, including empathy, toward artificial entities. People may feel a sense of empathy toward cleaning robots, pet robots, and conversational agents used in e-commerce and customer support. However, not all individuals accept artificial agents equally; some experience discomfort or resistance toward them [8,9].

Several studies have examined the relationship between trust and empathy in human-agent interaction. Spitale *et al*. [10] found that a social assistance robot elicited higher empathy when the story it narrated aligned with the user’s emotional focus. Similarly, Johanson *et al*. [11] showed that healthcare robots employing verbal empathetic statements and nonverbal cues significantly improved user trust and satisfaction. Tsumura and Yamada [12] investigated how an agent’s empathic behavior influenced trust recovery, demonstrating that successful interactions that included empathic expressions fostered increased trust.

Despite the potential for positive engagement, human responses to artificial agents are not always straightforward. Mori’s uncanny valley hypothesis [13] suggests that as robots become more human-like, they initially become more relatable, but beyond a certain threshold, they evoke discomfort. Similarly, Thepsoonthorn *et al*. [14] found that when nonverbal behaviors of robots became too human-like, they were perceived negatively, highlighting the complexity of human-agent relationships.

Another challenge in AI acceptance is algorithm aversion—the reluctance to trust decisions made by artificial systems. Mahmud *et al*. [15] conducted a literature review on algorithm aversion, noting that people tend to distrust algorithmic decisions, particularly when the stakes are high. Filiz *et al*. [16] further demonstrated that as the importance of a decision increases, algorithm aversion becomes more pronounced. These findings suggest that fostering positive relationships with AI is critical for their broader acceptance in society.

Empirical research on human-human interaction suggests that active participation in a task can enhance empathy and perspective-taking. For instance, Shaffer *et al*. [17] found that engaging in narrative writing increased positive attitudes toward individuals engaged in controversial behaviors. Drawing from these insights, we explore whether a similar mechanism applies to human-agent interaction—specifically, whether active involvement in creating an agent fosters empathy toward it.

One psychological phenomenon that may be relevant in this context is the IKEA effect, where individuals place greater value on self-assembled objects [18]. The IKEA effect has been extensively studied in consumer psychology, demonstrating that individuals who invest effort in constructing an item develop a stronger attachment to it. However, its potential application in human-agent interaction—specifically, its role in fostering empathy toward artificial agents—has not been explored.

Recent neuroscience research has begun to reveal the underlying mechanisms of the IKEA effect, emphasizing the roles of memory, attachment, and motivational systems in effort-based valuation. Using fNIRS, Oishi *et al*. [19] demonstrated increased activation in the left inferior and middle frontal gyri during evaluations of self-assembled products, suggesting enhanced memory and emotional engagement. Complementing this, Liu *et al*. [20] showed that participants exerted more effort to save their own decorated items, accompanied by stronger activation in the nucleus acumens and motor-motivational areas. These findings imply that the act of creating something enhances emotional attachment through reward-related neural circuits.

While many previous studies have explored empathy through socially expressive or interactive agents, we intentionally employed a non-interactive, pre-programmed LEGO Mindstorms EV3 robot to isolate the effects of user-initiated construction effort. This design enables us to control for confounding variables such as agent responsiveness or verbal expressivity—common in prior empathy studies but potentially masking the contribution of physical engagement and perceived effort.

In contrast to those studies that induce empathy through narrative or agent-driven behavior, our approach tests whether the IKEA effect—operationalized through the physical act of assembling a robot—can serve as a bottom-up mechanism for empathy formation. By embedding the IKEA effect into a realistic construction task, this study bridges psychological theory and practical agent design.

Building on these previous studies, we hypothesize that the IKEA effect can enhance human empathy toward artificial agents, leading to a more positive relationship between humans and AI. As agents become increasingly embedded in social and professional settings, fostering stronger emotional connections between humans and agents may improve user acceptance and engagement.

This study aims to investigate how active engagement in assembling an artificial agent influences empathy toward that agent. Specifically, we examine the following questions:

1. Whether the IKEA effect enhances empathy toward artificial agents.
2. Whether cooperative versus individual assembly affects empathy levels.
3. Whether empathy toward agents differs from empathy toward human co-participants.

By addressing these questions, we aim to provide insights into how user participation in the creation of artificial agents influences their perception and emotional connection to them. Our findings may contribute to the design of interactive technologies that promote better human-agent relationships and broader AI acceptance.

## Related work

### Definition of empathy

Empathy plays a crucial role in fostering social acceptance of AI agents. In human society, empathy facilitates interpersonal relationships by enabling individuals to understand and share the emotions of others [21,22]. Previous studies have provided general definitions and classifications of empathy in psychology, which have been widely applied in engineering and HRI studies.

Empathy has been categorized into different types in psychological research. Omdahl [23] identify empathy into three types: (1) affective empathy, which refers to emotional responses to others’ emotions, (2) cognitive empathy, which involves understanding the emotional state of others, and (3) comprehensive empathy, which includes both affective and cognitive components. Similarly, Preston and de Waal [24] proposed the perception-action model (PAM) and unified different perspectives on empathy. They defined three aspects of empathy: (a) sharing or being influenced by others’ emotional states, (b) assessing the reasons behind emotions, and (c) incorporating other perspectives. Arbel *et al*. [25] expanded on existing classification by introducing the concept of adaptive empathy, which refers to the ability to learn and adjust one’s empathic response based on feedback.

These foundational classifications not only provide a basis for psychological understanding, but also clarify the dimensions of empathy that may be affected by specific interventions, such as agent assembly. In particular, our study focuses on affective and cognitive empathy, two dimensions likely to be differentially influenced by physical construction tasks.

Although our study focuses on the positive role of empathy in enhancing societal acceptance of AI agents, psychological literature also discusses its negative effects. Bloom [26] highlighted that empathy, while often considered a moral guide, can also lead to irrational decision-making, favoritism, and even aggression. He argued that overcoming these biases requires conscious, deliberative reasoning and altruistic approaches.

Empathy is commonly measured using standardized questionnaires. The Interpersonal Reactivity Index (IRI) is widely used in psychology to assess various aspects of empathy [27]. Baron-Cohen and Wheelwright [28] developed the Empathy Quotient (EQ) as a self-report measure of empathy in adults. Lawrence *et al*. [29] examined the reliability and validity of EQ, finding moderate correlations between its subscales and those of IRI. Among these, IRI has been extensively used in psychology, as well as in HRI and HAI research [17,30,31], to investigate how empathy influences human perception of artificial agents.

These scales provide essential tools for operationalizing empathy in empirical settings. As we investigate whether constructing an agent can alter participants’ empathy, selecting an instrument that can differentiate between cognitive and affective aspects of empathy was critical to aligning our measures with the theoretical constructs discussed above.

Given that empathy significantly impacts trust and acceptance of AI agents, we adapted the Interpersonal Reactivity Index (IRI) to measure empathy toward agents as a key measurement tool in this study. By applying psychological theories of empathy to the field of HRI/HAI, we aim to deepen our understanding of human-agent relationships and explore ways to enhance agent acceptance through empathetic interactions.

### Empathy in engineering

Empathy has also been explored within engineering domains, particularly through the use of immersive technologies such as virtual reality (VR). van Loon *et al*. [32] examined whether taking another’s perspective in VR environments could enhance empathic responses, especially when participants interacted with avatars that resembled their assigned roles. Their results indicated that perspective-taking was more effective when there was a strong alignment between the avatar and the participant’s assumed identity. Building on this, Herrera *et al*. [33] conducted a comparative study of traditional versus VR-based empathy interventions, assessing both immediate and lasting effects. They further investigated how varying degrees of immersion influenced the emotional and cognitive components of empathy, depending on the mediating technology employed.

Empathy in VR has also been studied in the context of social bias reduction. Tassinari *et al*. [34] designed an inter-participant study to examine how VR can facilitate positive intergroup contact with members of marginalized communities, particularly ethnic minorities. Their findings demonstrated that intergroup interactions in VR could enhance empathy toward out-group members. Crone and Kallen [35] investigated the influence of perspective-taking tasks conducted through online platforms and immersive VR on reducing gender bias. Their findings indicated that VR-based interventions produced a more immediate shift in participants’ behavior compared to text-based online conditions, which was attributed to the enhanced sense of presence and engagement that immersive environments typically afford.

These VR-based studies demonstrate how perspective-taking mechanisms—particularly when embodied or effortful—can activate empathy even in mediated or artificial settings. This concept is foundational to our study, which aims to investigate whether physical engagement with an agent (through assembly) can likewise trigger similar empathic responses. In both cases, the user’s active role appears central to empathy induction.

Beyond VR applications, empathy is also gaining attention in product and interaction design. Bennett and Rosner [36] explored human-centered design processes that emphasize the “promise of empathy," wherein designers strive to deeply understand target users to improve technology development. Similarly, Rahmanti *et al*. [37] developed a chatbot named “SlimMe" that delivers motivational support through simulated empathic interactions in the context of dieting. Their research analyzed user engagement with the system and introduced a text-based emotional interpretation mechanism, allowing the chatbot to detect user affect and respond with contextually appropriate empathic expressions.

Empathy-driven design is also a growing focus in service and AI interactions. Drouet *et al*. [38] developed an empathy scale tailored for service design, highlighting the often-overlooked role of fostering user empathy in delivering high-quality, user-centered services and products. Al-Farisi *et al*. [39] examined how anthropomorphic design cues (ADCs), including verbal and nonverbal elements, influence chatbot interactions. Their findings showed that ADCs significantly enhanced users’ perception of the chatbot’s empathic capabilities.

Across these studies, empathy is shown not merely as a trait of systems but as an emergent property of the interaction between human and system—whether through embodiment, narrative, or personalization. Our experimental design aligns with this view by testing whether self-driven interaction with a non-social system (a programmable LEGO agent) can still elicit empathic responses through a mechanism rooted in user effort and ownership.

These studies demonstrate that empathy is a critical factor in various engineering applications, from virtual reality to human-centered design and AI interactions. As technology continues to advance, incorporating empathic elements into engineering and design processes may lead to more effective and socially accepted interactive systems.

### Empathy in human-robot/agent interaction

Studies in the field of human-robot interaction (HRI) have explored how humans empathize with artificial agents. Yamada and Komatsu [40] proposed a design policy called “SE2PM: simple expression to primitive mind" using a mobile robot that expresses its internal state through beeps and a pet robot, AIBO, that conveys mental expressions through complex behaviors. Their study demonstrated that intuitive, simple expressions, such as beeps from basic robots, are more effective in conveying a robot’s primitive mind to humans compared to more complex behaviors exhibited by sophisticated robots.

Building upon the concept of cognitive developmental robotics, Asada [41] proposed the framework of “affective developmental robotics," which aims to generate more genuine expressions of artificial empathy. In this framework, artificial empathy refers to AI systems—such as companion robots and virtual agents—that can perceive human emotions and produce empathic responses accordingly. The implementation of artificial empathy is regarded as a core element of social robotics, playing a key role in facilitating emotionally resonant interactions that support the societal integration of robotic agents.

Fraune [42] examined how individuals’ moral judgments and perceptions of agents are influenced by factors such as social group alignment (in-group vs. out-group), the nature of the agent (human vs. robot), and the level of anthropomorphism (anthropomorphic vs. mechanistic). The findings indicated that robots with anthropomorphic features elicited more favorable evaluations than mechanistic ones, reflecting patterns commonly observed in human interpersonal interactions. Park and Whang [43] conducted a systematic review of empathy research in interpersonal interactions, virtual agents, and social robots. They identified key design factors such as domain dependence, multimodality, and empathic coordination, which should be considered when developing empathic social robots.

In the field of human-agent interaction (HAI), Leite *et al*. [44] carried out a longitudinal study in an elementary school setting to assess the impact of an empathy-driven model implemented in social robots engaging with children. Their results indicated that this model enhanced children’s sense of social presence, increased their engagement, and fostered perceptions of social support during repeated interactions. Chen and Wang [45] proposed a unified model of empathy and anti-empathy, suggesting that both are influenced by an entity’s perceived coexistence and competition within a group. They introduced the Adaptive Empathetic Learner (AEL), an agent training framework designed to evaluate and optimize empathetic responses in multi-agent systems.

Perugia *et al*. [46] examined how personality traits and empathy influence facial mimicry in interactions between humans and artificial agents. Their study found that mimicry was significantly affected by the agent’s embodiment but not by its perceived humanness. Additionally, individual differences in sociability, empathy, and emotion recognition influenced mimicry responses. Parmar *et al*. [47] systematically analyzed the effects of animation quality, voice quality, rendering style, and simulated empathy on virtual agents’ perceived naturalness, engagement, trust, credibility, and persuasiveness in healthcare applications.

Tsumura and Yamada [48] examined human empathy as a key attribute for agent acceptance and proposed the concept of empathy agents designed to foster human-agent relationships. They later explored the role of self-disclosure in anthropomorphic agents, demonstrating that self-disclosure from agents to humans can enhance human empathy toward the agent [30]. Their subsequent work investigated various factors influencing human empathy toward agents, including task-based interactions and agent behavior patterns [31]. Additionally, they examined how agent reactions and human preferences affect empathy and acceptance of agent mistakes, revealing that agent mistakes reduce empathy but are tolerated under certain conditions [49]. Further studies focused on self-disclosure attributes and their impact on human-agent relationships, finding that while self-disclosure did not significantly influence trust, it did affect human empathy toward agents [50].

Across these studies, a recurring theme is that empathy toward agents is modulated by agent appearance, interactivity, emotional expressivity, and task-based engagement. However, many existing studies rely on interactive or social agents that explicitly express emotion. Our study departs from this by investigating whether empathy can also emerge toward passive, non-expressive agents, specifically through self-assembly effort.

To clarify the nature of empathy in human-agent interactions, Paiva *et al*. [51] proposed a dual model of empathy agents, distinguishing between agents that target empathy (eliciting empathy from humans) and those that empathize with observers. In this study, we adopt Paiva’s framework, considering the agent as an object of empathy and examining how human participants’ empathetic responses are shaped in interaction with artificial agents.

This approach allows us to position our investigation within the broader HRI/HAI landscape while uniquely focusing on user-initiated effort as a mechanism for empathy induction—thus connecting the literature on empathic agents with psychological constructs such as the IKEA effect.

### IKEA effect and human relationship

Sun and Sundar [52] investigated whether assembling a robot enhances the quality of interaction with the robot and whether it matters whether the robot is perceived as a practical tool or as a socially interactive entity. Their results indicated that when participants set up the robot themselves, they tended to have more positive evaluations of both the robot and the interaction process. These effects were positively mediated by a sense of ownership and accomplishment, while they were negatively mediated by perceived process costs.

Marsh *et al*. [53] examined the developmental stages of the IKEA effect, demonstrating that this cognitive bias emerges at around five years of age, a key developmental milestone in the formation of self-concept. They also assessed the role of effort in the effect, finding that the amount of effort expended did not moderate the IKEA effect in five- to six-year-olds. Furthermore, they investigated whether feelings of ownership explained the effect and found that ownership alone did not fully account for why children valued their own work more highly.

These findings support the notion that task-based physical engagement can enhance subjective evaluation of artifacts through mechanisms such as ownership and self-concept reinforcement—mechanisms also hypothesized to drive empathy formation in our current study. In particular, our investigation extends this line of work by examining whether such value inflation via the IKEA effect also manifests in empathic evaluations of artificial agents.

Wald *et al*. [54] explored the potential of user-based chatbot customization in developing trust. While customization itself had no direct impact on trust, the study identified anthropomorphism as an important mediating factor. Aeschlimann *et al*. [55] analyzed communication patterns and prosocial outcomes in interactions with voice assistants. Their findings indicated that children did not hold the same expectations for voice assistants as they did for human interlocutors, highlighting differences in human-to-human and human-to-computer cooperation.

These studies suggest that social perceptions of agents are shaped not only by interactive features but also by the nature of engagement and role attribution. Our work leverages a non-social robotic platform—LEGO Mindstorms EV3—to test whether empathy can emerge purely from construction effort, without relying on anthropomorphic expressivity or social framing.

Velentza *et al*. [56] applied participatory design procedures to identify user attitudes and needs in educational settings and developed the STIMEY robot based on these insights. Their cross-European study revealed that students positively evaluated the interactive capabilities of the robot and its ability to provide feedback. Additionally, statistical analysis showed that students’ attitudes toward the usability of the robot improved significantly after engaging with it in lessons.

Pauw *et al*. [57] examined the socio-emotional benefits of interacting with virtual humans and whether these benefits varied depending on the type of support provided. They compared emotional and cognitive support across two emotional states (anger and worry), and results showed that participants experienced reduced emotional intensity and improved mood after the interaction.

Mott *et al*. [58] conducted co-design workshops with elementary school students from different age groups at a forest school in Denver, Colorado. Their study examined how children’s understanding of robot technology influences their critical thinking regarding ethical dilemmas in child-robot relationships.

Spaccatini *et al*. [59] experimentally investigated whether the type of mind attributed to anthropomorphic social robots has a complementary effect on empathy for people in need. Their results indicated that anthropomorphism facilitated the attribution of subjectivity when the robot engaged in chatbot-based interactions, and the attribution of experience when it had an anthropomorphic appearance. Jorge *et al*. [60] studied the impact of automation failures in human-automation collaborative scenarios on human confidence in automation. Their findings demonstrated that automation failures negatively influenced both human trust in automation and their overall perception of automated systems.

Together, these studies underscore the importance of perceived agency, customization, and shared experiences in shaping affective responses toward artificial systems. Our study adds a new dimension to this conversation by testing whether non-interactive, agent-directed physical effort—specifically self-assembly—can elicit empathy in the absence of socially rich cues. By situating the IKEA effect in the domain of empathy rather than just perceived utility or trust, our work expands the conceptual applicability of construction-based engagement in human-agent interaction.

## Materials and methods

### Ethics Statement

The protocol was approved by the ethics committee of the National Institute of Informatics (No. 1, 13, April, 2020). All studies were carried out in accordance with the recommendations of the Ethical Guidelines for Medical and Health Research Involving Human Subjects provided by the Ministry of Education, Culture, Sports, Science and Technology and Ministry of Health, Labour and Welfare in Japan. Written informed consent was provided by choosing one option on an online form: “I am indicating that I have read the information in the instructions for participating in this research. I consent to participate in this research." All participants gave informed consent. After that, they were debriefed about the experimental procedures. The experiment was conducted from 22 to 28 May 2023 (Japan time).

### Hypotheses

The purpose of this study is to examine whether assembling an agent induces the IKEA effect and enhances human empathy toward the agent. Additionally, we investigate whether the social relationship between participants during the task influences their empathy toward both the agent and their partner. To address these objectives, we used LEGO Mindstorms EV3 and measured participants’ empathy for both the agent and their co-participant before and after the task. Based on prior research and theoretical considerations, we propose the following three hypotheses:

**H1:** Assembling an agent promotes empathy toward the agent through the IKEA effect.
**H2:** Cooperative assembly increases empathy toward the co-participant, but less than toward the self-assembled agent.
**H3:** When participants assemble an agent individually, their empathy toward the agent is greater than their empathy toward the co-participant.

H1 is based on the notion that the IKEA effect influences individual’s perception of value, making them more likely to attribute emotional significance to self-assembled entities. H2 and H3 are derived from previous findings suggesting that collaborative tasks enhance interpersonal empathy [42,52,57]. It is also plausible that the target of empathy (agent or co-participant) is influenced by the nature of the task relationship.

To test these hypotheses, we designed a mixed-design experiment with three factors: the IKEA effect, participant relationships, and the empathy object. Each factor had two levels: the IKEA effect (before vs. after the task), participant relationships (cooperative vs. individual), and empathy object (agent vs. co-participant). Participant relationships were treated as a between-participants factor, while the IKEA effect and empathy object were within-participants factors. The dependent variable was the level of empathy reported by participants. Participants were randomly assigned in pairs to either the cooperative or individual condition upon recruitment to ensure unbiased group allocation.

### LEGO Mindstorms EV3

**Fig 1:**
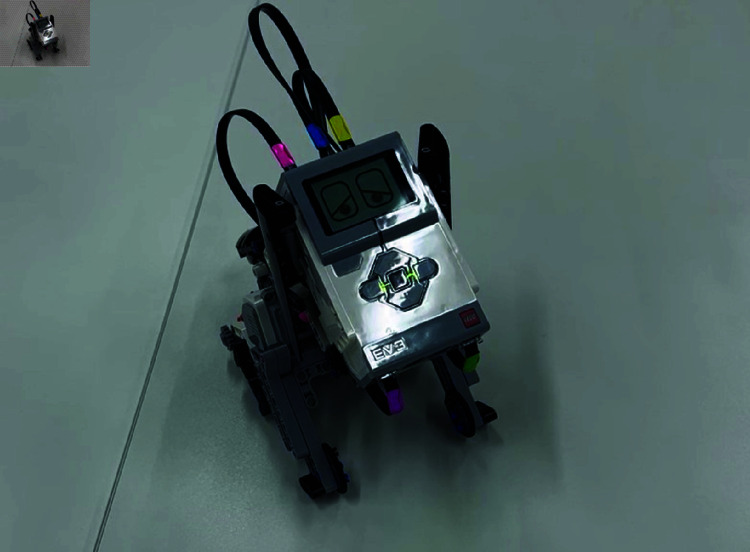
LEGO agent.

The LEGO Mindstorms EV3 (“LEGO Agent") used in this study is an assemblable robot. The participants assembled a dog-shaped robot during the experiment, one of the possible forms of LEGO Mindstorms EV3. Fig 1 shows the finished robot assembled in the task.

**Fig 2:**
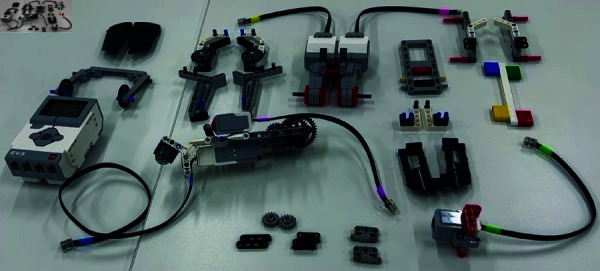
Parts before assembly.

Although its behavior can be controlled by programming, programming was not required for this study, so participants only had to confirm that the sample program worked successfully after the assembly was completed. In this experiment, to simplify the assembly process, we assembled the small parts of the robot in advance, and the finished product shown in Fig 2 was assembled from its disassembled state. Information on this LEGO agent is summarized below.

**Model number**: EVR45544S
**Weight**: 791 g
**Height**: 17.2 cm
**Average assembly time**: 45 minutes

### Experimental procedure

**Fig 3:**
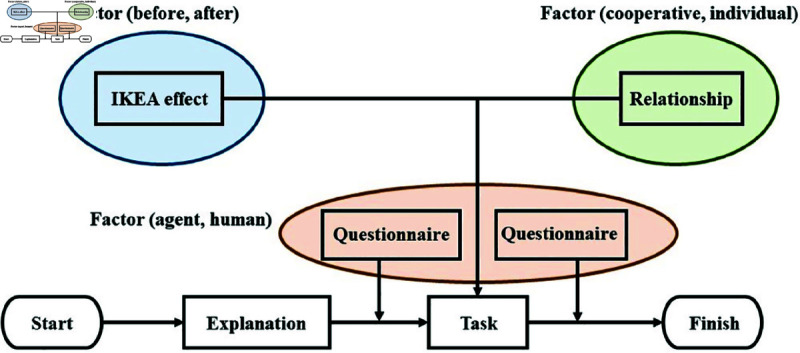
Process flow of the experiment.

**Fig 4:**
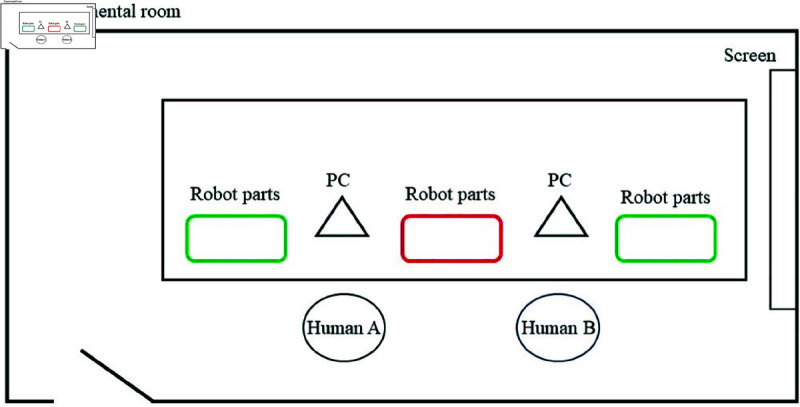
Experimental environment setup.

This experiment was conducted at the National Institute of Informatics, with participants recruited through a recruitment website. The procedure and environment are outlined below. The experimental procedure is outlined in Fig 3, and the experimental environment is illustrated in Fig 4. The following explanation is based on Fig 4.

Before starting the assembly task, participants were shown a video demonstrating the fully assembled LEGO agent, which had been pre-constructed by the experimenters. This was to ensure that participants understood what they were expected to create. Participants were seated in positions A and B and were assigned either a cooperative or individual task condition. In the cooperative condition, participants worked together to assemble a single LEGO agent. The necessary parts were placed in red-marked locations, requiring collaboration. In the individual condition, each participant assembled their own LEGO agent independently. The parts were placed in green-marked locations, ensuring separation of materials.

A computer was provided in front of each participant to display the assembly instructions and administer surveys. Participants were allowed to communicate freely throughout the assembly process, as prohibiting conversation would not reflect a natural collaborative environment. Participants were instructed to assemble a dog-shaped LEGO agent using LEGO Mindstorms EV3. Those in the cooperative condition built a single agent together, while those in the individual condition each built their own agent separately. The assembly process was conducted in a designated experimental room, and participants remained in the room for the duration of the task. The total experiment duration was approximately one hour.

The core focus of this experiment was to explore how assembling an agent affects empathy toward the agent (the IKEA effect), and whether the relationship between participants modulates their empathy toward each other. After completing the assembly, participants were given a few minutes to observe and interact with the LEGO agent to confirm that it functioned as expected. This interaction period lasted approximately 5 minutes for all participant pairs and was kept consistent across conditions. The timing was monitored by the experimenter to ensure uniformity.

This interaction phase was designed to reinforce the sense of completion and ownership, as the agent’s behavior was identical to what was shown in the initial demonstration video. The specific behaviors of the agent can be seen in the supplementary video materials (S1 Video). Following this, participants completed the same questionnaire they had taken before the task to assess changes in their empathy toward both the agent and their co-participant.

### Participants

Through the recruitment website, participants were paid 1,500 yen (= 10.27 dollars). The number of participants was 40. To ensure that the number of participants was sufficient for the statistical analysis of the three-factor mixed design, we used G*power, a software tool that can check the appropriate number of participants for statistical analysis [61]. A power analysis was conducted using GPower to determine the minimum required sample size for a 2×2×2 mixed-design ANOVA (F tests, repeated measures, within-between interaction). The analysis indicated that an adequate sample size was 24 participants. To enhance the robustness of the findings and account for potential variability or dropout, we recruited a total of 40 participants, assigning 20 participants to each group. GPower settings: F tests, ANOVA: Repeated measures, within-between interaction; Effect size f = 0.25; α error probability = 0.05; Power (1–β error probability) = 0.8; Number of groups = 2; Number of measurements = 4; Correlation among repeated measures = 0.5; Nonsphericity correction ϵ = 1.

Cronbach’s α coefficient was then applied to the 40 participants to determine the reliability of their survey responses and found to be between 0.723 and 0.890 in all conditions. Twenty participants in each condition were included in the analysis. The mean age was 26.38 years (standard deviation 7.693), with a minimum of 18 years and a maximum of 52 years. Gender was 25 males and 15 females. The full dataset used in the analysis is provided in the Supporting Information (S1 File).

### Questionnaire

Since the results of previous studies have shown that the EQ scale is related to the IRI scale, we used the IRI questionnaire [27], which has fewer questions and allows for the four characteristics of empathy to be investigated. We also used the widely used IRI for comparison with previous and future research on empathy. We also focused on investigating the impact of each characteristic of empathy. The use of the IRI was appropriate for investigating this as based on previous studies.

Participants answered questionnaires before and after the task. This was a 12-item questionnaire modified from the IRI. As the IRI was originally designed to investigate human empathy characteristics, we modified it to apply to both the artificial agent and the co-participant by replacing the generic term “character” with either “LEGO agent” or “co-participant.” This adaptation approach has been used in prior studies on empathy toward robots and agents [30,31]. The effectiveness of this modification was confirmed by calculating Cronbach’s alpha for each condition, which ranged from 0.723 to 0.890, indicating acceptable to high reliability. We believe this supports the internal consistency and contextual suitability of our modified scale for measuring empathy in both human-human and human-agent interactions.

While the original IRI includes four subscales, we structured our modified version around two empirically validated categories: affective empathy (Personal Distress, Empathic Concern) and cognitive empathy (Perspective Taking, Fantasy Scale). This allows alignment with the theoretical framework employed in our analysis.

The part of the description that says “character" changed between LEGO agent or other participant. The same questionnaire was applied both before and after the task and was administered on a 5-point Likert scale (1: not applicable, 5: applicable), as shown in Table 1. Q4, Q9, and Q10 are inverted items, so the scores were reversed when analyzing them. Q1 to Q6 examine affective empathy, and Q7 to Q12 examine cognitive empathy.

**Table 1:** Modified empathy questionnaire based on IRI: subscales and item descriptions [30,31].

Affective empathy
**Personal distress**
Q1: If an emergency happens to the character, you would be anxious and restless.
Q2: If the character is emotionally disturbed, you would not know what to do.
Q3: If you see the character in need of immediate help, you would be confused and would not know what to do.
**Empathic concern**
Q4: If you see the character in trouble, you would not feel sorry for that character.
Q5: If you see the character being taken advantage of by others, you would feel like you want to protect that character.
Q6: The character’s story and the events that have taken place move you strongly.
**Cognitive empathy**
**Perspective taking**
Q7: You look at both the character’s position and the human position.
Q8: If you were trying to get to know the character better, you would imagine how that character sees things.
Q9: When you think you’re right, you don’t listen to what the character has to say.
**Fantasy scale**
Q10: You are objective without being drawn into the character’s story or the events taken place.
Q11: You imagine how you would feel if the events that happened to the character happened to you.
Q12: You get deep into the feelings of the character.

### IKEA effect

The IKEA effect refers to the phenomenon in which individuals place greater value on self-made objects due to the effort invested in their creation [18]. This study builds on the theoretical premise that such effort-driven valuation can also extend to artificial agents, potentially enhancing empathy toward them. To operationalize the IKEA effect, participants assembled a dog-shaped LEGO Mindstorms EV3 robot, a method consistent with prior IKEA effect studies [18]. Unlike previous research that focused on ownership or monetary valuation, this study explores whether effort investment enhances emotional and cognitive empathy toward agents.

By assessing empathy levels before and after the assembly task, we aim to determine whether this self-construction process leads participants to relate more closely to the agent—not merely as a tool, but as a socially meaningful entity. In order to simplify the assembly process while maintaining participant engagement, some smaller components of the robot were pre-assembled by the experimenters. As shown in Fig 2, participants assembled the robot from a partially disassembled state. The assembly process was guided by a pre-recorded instructional video, which participants followed while constructing the LEGO agent.

**Fig 5:**
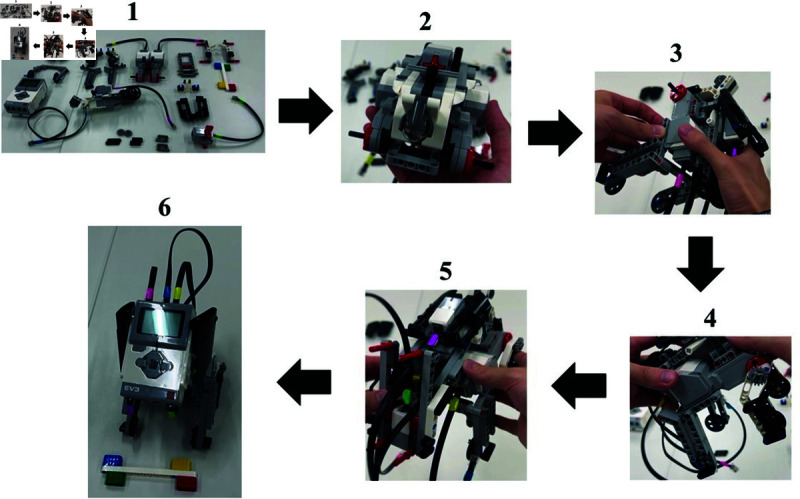
Specific procedure for assembling experiment.

The assembly procedure remained consistent across both cooperative and individual conditions, taking approximately 45 minutes on average. The step-by-step assembly process is illustrated in Fig 5. Participants first assembled the fuselage, followed by attaching the front legs, then the back legs, and subsequently the neck. Finally, the head was attached to complete the assembly. By structuring the experiment in this manner, we aimed to examine whether assembling the agent would induce the IKEA effect and influence participants’ empathy toward the agent.

### Participants’ relationships

This study examines how different participant relationships during the assembly task influence empathy toward the agent and the co-participant. Participants were assigned to one of two conditions: a cooperative assembly condition or an individual assembly condition.

**Fig 6:**
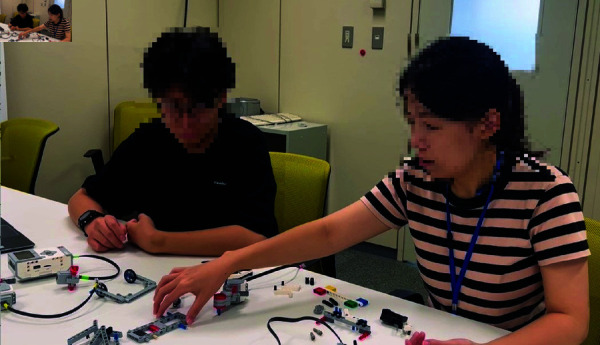
Participants cooperating to assemble a single LEGO agent during the experiment.

In the cooperative condition, two participants worked together to assemble a single LEGO agent while watching the assembly video. They were encouraged to communicate and check each other’s progress throughout the task. As shown in Fig 6, this cooperative setting fostered interaction between participants, which may have influenced their relationship. However, how this cooperation affected their empathy toward the assembled agent remained unclear.

**Fig 7:**
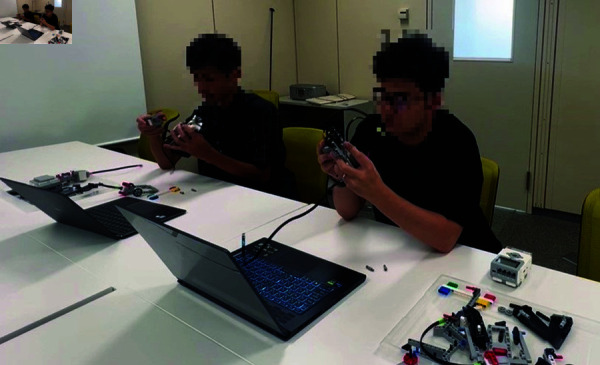
Participants independently assembling LEGO agents in the individual condition of the experiment.

In the individual condition, each participant independently assembled their own LEGO agent while watching the assembly video. However, both participants were in the same room and performed the task simultaneously. This setup, illustrated in Fig 7, was designed to explore whether merely sharing a workspace would impact their empathy toward the co-participant, even in the absence of direct collaboration.

In both conditions, participants could watch the assembly video as needed and were not restricted from talking to each other. This ensured that natural interactions could take place, allowing us to assess the impact of both collaborative and non-collaborative settings on empathy toward the agent and the co-participant.

We acknowledge that physical co-presence may have influenced participants’ perceptions; however, separating participants into entirely isolated spaces would have undermined the ecological validity and central aim of this study—to examine empathy improvement in human-agent interactions situated in naturally social contexts. To reduce the influence of interaction, participants in the individual condition used physically separated materials (green-marked areas) in Fig 4, followed individual instructions, and were not required or encouraged to interact. This design choice allowed us to simulate realistic settings such as classrooms or workshops, where individual tasks often occur in shared spaces.

### Empathy object

This study investigates two distinct empathy objects: the artificial agent (LEGO robot) and the human co-participant. While previous research has often focused on either human-agent empathy or human-human empathy, direct comparisons within a shared task context remain rare. Our approach provides a novel perspective by placing both targets within the same interactional frame, allowing us to explore how empathy may be differentially elicited by artificial versus human entities.

The inclusion of both targets enables us to assess the relative strength of empathy induced by self-assembly (IKEA effect) versus social interaction. Specifically, we examine whether participants show greater empathy toward the self-assembled agent—reflecting emotional attachment derived from effort investment—or toward their peer, reflecting interpersonal engagement.

This dual-target comparison is grounded in the media equation theory [1], which posits that people tend to respond to media and artifacts in socially meaningful ways. However, the degree to which empathy is transferred to artificial agents, particularly in comparison to human partners, remains underexplored. Our study addresses this gap by directly evaluating how empathy is distributed across human and non-human entities in a controlled but socially situated task environment.

### Analysis method

The analysis was a three-factor mixed design ANOVA. The between-participants factor consisted of two levels of participant relationships (cooperative and individual). The within-participant factor consisted of two levels before and after the IKEA effect and the empathy object (agent, another participant) empathy value.

On the basis of the results of the participants’ questionnaire responses, we investigated the influence of the IKEA effect and the participants’ relationships as factors eliciting empathy for the agent and empathy for the other participant. The empathy values before and after the task were used as the dependent variable. R (ver. 4.1.0) was used for ANOVA.

## Results

This section reports the results of the questionnaire data analyzed using a three-way mixed ANOVA. Empathy was classified into two subtypes: affective empathy and cognitive empathy. Descriptive statistics for each condition are provided in Table 2, and the raw data are available in the Supplementary Information.

**Table 2:** Descriptive statistics of empathy scores: Mean and S.D. across conditions, empathy types, and targets (agent vs. human).

Category		Conditions	Mean	S.D.
Empathy (Q1-Q12)	before	cooperative-agent	35.00	7.455
		cooperative-human	44.60	6.707
		individual-agent	34.70	10.04
		individual-human	42.20	6.748
	after	cooperative-agent	38.95	9.185
		cooperative-human	43.25	6.851
		individual-agent	37.85	8.744
		individual-human	43.40	5.716
Affective empathy (Q1-Q6)	before	cooperative-agent	18.75	3.338
		cooperative-human	22.60	3.560
		individual-agent	17.80	5.064
		individual-human	21.05	4.174
	after	cooperative-agent	20.10	3.986
		cooperative-humans	22.20	4.491
		individual-agent	18.85	4.815
		individual-human	21.60	3.952
Cognitive empathy (Q7-Q12)	before	cooperative-agent	16.25	4.993
		cooperative-human	22.00	3.880
		individual-agent	16.90	5.360
		individual-human	21.15	3.937
	after	cooperative-agent	18.85	5.697
		cooperative-human	21.05	3.576
		individual-agent	19.00	4.812
		individual-human	21.80	2.628

The distinction between affective and cognitive empathy is theoretically grounded: affective empathy refers to emotional resonance with others’ experiences, while cognitive empathy involves understanding others’ mental states and perspectives. These subtypes may respond differently to the IKEA effect due to their underlying mechanisms. Particularly, cognitive empathy is more likely to be modulated by task-based engagement and perspective-taking, which are central to self-assembly experiences.

**Fig 8:**
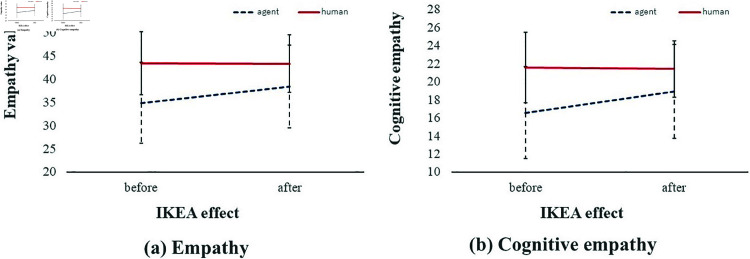
Interaction results for (a) empathy and (b) cognitive empathy.

The ANOVA results (Table 3) revealed a significant interaction between the IKEA effect and the empathy object (agent vs. co-participant), as illustrated in Fig 8. In contrast, no significant interactions were observed between participant relationship and either the IKEA effect or the empathy object.

**Table 3:** Three-way ANOVA results: effects of IKEA effect, participant relationship, and empathy object.

	Factor	*F*	*p*	$\eta_{\text{p}}^{2}$
Empathy (Q1-12)	Participants’ relationships	0.266	0.609 *ns*	*0.007*
	IKEA effect	5.451	0.025 *	0.125
	Empathy object	24.44	0.000 ***	0.391
	Participants’ relationships × IKEA effect	0.346	0.560 *ns*	*0.009*
	Participants’ relationships × Empathy object	0.024	0.877 *ns*	*0.001*
	IKEA effect × Empathy object	5.989	0.019 *	0.136
	Participants’ relationships × IKEA effect × Empathy object	1.279	0.265 *ns*	*0.033*
Affective empathy (Q1-6)	Participants’ relationships	1.194	0.281 *ns*	*0.031*
	IKEA effect	2.003	0.165 *ns*	*0.050*
	Empathy object	20.17	0.000 ***	0.347
	Participants’ relationships × IKEA effect	0.130	0.720 *ns*	*0.003*
	Participants’ relationships × Empathy object	0.000	0.985 *ns*	*0.000*
	IKEA effect × Empathy object	2.333	0.135 *ns*	*0.058*
	Participants’ relationships × IKEA effect × Empathy object	0.720	0.402 *ns*	*0.019*
Cognitive empathy (Q7-12)	Participants’ relationships	0.034	0.854 *ns*	*0.001*
	IKEA effect	6.686	0.014 *	0.150
	Empathy object	19.85	0.000 ***	0.343
	Participants’ relationships × IKEA effect	0.418	0.522 *ns*	*0.011*
	Participants’ relationships × Empathy object	0.072	0.791 *ns*	*0.002*
	IKEA effect × Empathy object	7.354	0.010 **	0.162
	Participants’ relationships × IKEA effect × Empathy object	1.297	0.262 *ns*	*0.033*

p: *p < 0.05 **p < 0.01 ***p < 0.001

Since the central hypotheses of this study concern how the IKEA effect modulates empathy toward different targets (agents vs. humans), identifying interaction effects is critical for validating our theoretical framework. Given the presence of such interactions, we focus on simple main effect analyses rather than reporting main effects independently. The results of these analyses are summarized in Table 4.

**Table 4:** Simple main effects analysis: IKEA effect and empathy object comparisons.

	Factor	*F*	*p*	$\eta_{\text{p}}^{2}$
Empathy (Q1-12)	IKEA effect in empathy for agent	6.947	0.012 *	0.155
	IKEA effect in empathy for human	0.014	0.905 *ns*	*0.000*
	Empathy object before IKEA	29.07	0.000 ***	0.434
	Empathy object after IKEA	10.55	0.002 **	0.217
Cognitive empathy (Q7-12)	IKEA effect in empathy for agent	9.447	0.004 **	0.199
	IKEA effect in empathy for human	0.111	0.741 *ns*	*0.003*
	Empathy object before IKEA	25.31	0.000 ***	0.400
	Empathy object after IKEA	7.317	0.010 *	0.162

*p*: *p < 0.05 **p < 0.01 ***p<0.001

### General empathy

Summary: This analysis revealed that empathy toward agents significantly increased after the assembly task, consistent with the IKEA effect and supporting H1. In contrast, empathy toward co-participants remained unchanged, providing no support for H2. Furthermore, the predicted dominance of agent-directed empathy in the individual condition (H3) was not observed.

The three-way ANOVA (Table 3) revealed a significant interaction between the IKEA effect and the empathy object (F(1,38)=5.989, *p* = 0.019, $\eta_{p}^{2}=\mathrm{.136}$). A simple main effect analysis (Table 4) confirmed that empathy toward the agent increased significantly after assembly (*p* = 0.012, $\eta_{p}^{2}=\mathrm{.155}$), while no change was observed for empathy toward the human co-participant (*p* = 0.905).

A simple main effect analysis also revealed that empathy toward the human co-participant was significantly higher than that toward the agent, both before assembly (*p* < 0.001, $\eta_{p}^{2}=\mathrm{.434}$) and after assembly (*p* = 0.002, $\eta_{p}^{2}=\mathrm{.217}$). This suggests that although the IKEA effect enhanced empathy toward agents, the overall empathy level directed at co-participants remained substantially greater throughout the task.

**Fig 9:**
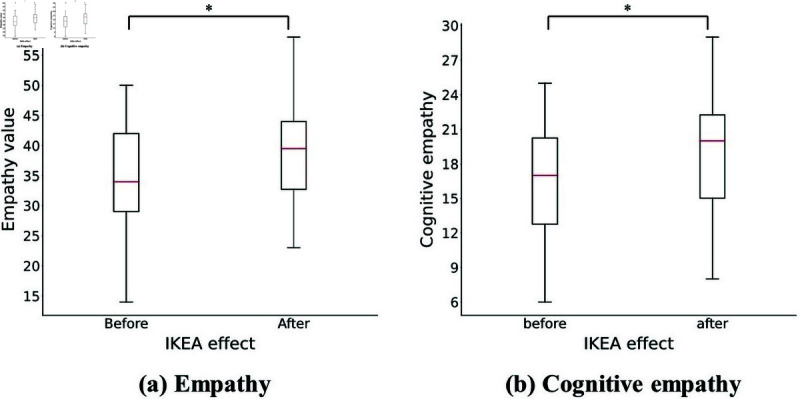
Results of post-task (a) empathy value and (b) cognitive empathy represented by box plots. Red lines are medians.

Specifically, empathy scores for agents increased from *Mean* = 34.85 (*S*.*D*. = 8.728) to *Mean* = 38.40 (*S*.*D*. = 8.869), whereas scores for human co-participants remained stable (before: *Mean* = 43.40, *S*.*D*. = 6.751; after: *Mean* = 43.33, *S*.*D*. = 6.228). Descriptive trends from Table 2 align with these statistical results. Fig 8(a) depicts this interaction, while Fig 9(a) presents box plots confirming the upward shift in agent-directed empathy and the stability of human-directed empathy.

These findings suggest that while assembling an agent enhances perceived empathy through effort-based attachment, it does not override the inherently higher empathy levels people feel toward other humans. This asymmetry underscores the robustness of interpersonal empathy and indicates that the IKEA effect, while effective, may only partially close the gap between human-agent and human-human empathy.

### Affective empathy

Summary: Affective empathy was higher for co-participants than for agents, but was not significantly influenced by the IKEA effect.

The three-way ANOVA (Table 3) revealed no significant interaction between the IKEA effect and the empathy object for affective empathy (F(1,38)=2.333, *p* = 0.135, $\eta_{p}^{2}=\mathrm{.058}$). Similarly, there was no significant main effect of the IKEA effect itself (*p* = 0.165). A significant simple main effect of the empathy object was found both before the task (*F* = 25.31, *p*<0.001, $\eta_{p}^{2}=\mathrm{.400}$) and after the task (*F* = 7.317, *p* = 0.010, $\eta_{p}^{2}=\mathrm{.162}$), indicating that affective empathy was consistently higher toward the human co-participant than toward the artificial agent across all conditions and time points.

Descriptive data in Table 2 support these findings: mean affective empathy scores for humans were higher than for agents in both the cooperative and individual conditions (cooperative: *Mean* = 22.40, *S*.*D*. = 4.005; individual: *Mean* = 21.33, *S*.*D*. = 4.022), and little change occurred after the assembly task (before: *Mean* = 21.83, *S*.*D*. = 3.959; after: *Mean* = 21.90, *S*.*D*. = 4.241).

These findings suggest that affective empathy, which reflects emotional resonance and concern, may require more than physical interaction or effort-based engagement to be elicited by artificial agents. Emotional affinity toward human partners appears more robust and less susceptible to short-term task manipulations like the IKEA effect. In contrast to cognitive empathy, affective empathy may be more dependent on pre-existing interpersonal cues such as facial expressions, voice, or shared history—elements that artificial agents often lack.

### Cognitive empathy

Summary: Cognitive empathy toward the agent significantly increased after assembly, indicating the role of the IKEA effect in promoting perspective-taking.

The three-way ANOVA (Table 3) revealed a significant interaction between the IKEA effect and the empathy object (F(1,38)=7.354, *p* = 0.010, $\eta_{p}^{2}=\mathrm{.162}$). Main effects for both the IKEA effect (*p* = 0.014) and the empathy object (*p* < 0.001) were also significant, but our focus remains on the interaction. A simple main effect analysis (Table 4) showed that cognitive empathy toward the agent increased significantly after the assembly task (before: *Mean* = 16.58, *S*.*D*. = 5.124; after: *Mean* = 18.93, *S*.*D*. = 5.206; *p* = 0.004, $\eta_{p}^{2}=\mathrm{.199}$). In contrast, empathy toward the co-participant remained stable (before: *Mean* = 21.58, *S*.*D*. = 3.882; after: *Mean* = 21.43, *S*.*D*. = 3.121; *p* = 0.741).

Fig 8(b) presents the interaction pattern between the IKEA effect and the empathy object for cognitive empathy, showing a clear post-task increase in agent-directed empathy. Box plots in Fig 9(b) visually confirm this upward trend, while scores for human-directed empathy remained largely unchanged.

These results indicate that cognitive empathy—reflecting perspective-taking and fantasy scale—can be significantly influenced by task-based engagement with artificial agents. Unlike affective empathy, cognitive empathy appears more malleable and susceptible to mechanisms such as the IKEA effect, possibly due to its reliance on cognitive processing rather than emotional resonance.

Notably, although empathy toward human co-participants remained consistently higher than empathy toward agents, the gap narrowed after the task. This pattern suggests that the IKEA effect facilitates an improvement shift in empathy, especially for artificial agents. While affective empathy remained stable across targets, cognitive empathy appears to reflect a more flexible dimension of social cognition, which can be enhanced even in interactions with non-human entities.

## Discussion

### Supporting hypotheses

Previous studies [21,22] suggest that fostering human empathy toward anthropomorphic agents is essential for building trust and improving human-agent relationships. Empathy toward agents plays a crucial role in their acceptance in society, and when agents can appropriately engage human empathy, they may become more widely integrated into human environments.

This study examined whether assembling an agent influences empathy toward the agent and the co-participant through the IKEA effect and participant relationships. The results supported H1, indicating that the IKEA effect significantly promoted empathy toward the agent. Importantly, empathy toward the co-participant remained unchanged before and after the IKEA effect, reinforcing the idea that the effect specifically enhances emotional attachment to artifacts rather than social relationships.

However, H2 and H3 were not supported. While previous studies have shown that cooperative work can enhance interpersonal relationships [42,52,57], the current findings indicate that cooperative assembly did not significantly affect empathy toward either the agent or the co-participant. These results suggest that while the IKEA effect increases the perceived value and emotional engagement with the agent, social interactions during the assembly process do not necessarily influence empathy levels.

One possible reason why H2 was not supported is the nature of the cooperative task. Although participants worked together to assemble a single agent, the interaction was limited to functional communication, and the short duration (approximately an hour) may not have been sufficient to foster meaningful interpersonal empathy. Moreover, participants were generally unfamiliar with each other, which could have constrained the depth of social bonding during the task.

As for H3, while we hypothesized that individual assembly would lead to greater empathy toward the agent than toward the co-participant, the fact that participants remained in the same room may have introduced subtle social facilitation effects. Even without collaboration, physical co-presence can enhance social awareness, potentially raising baseline empathy toward the co-participant and reducing the expected contrast.

### Influence of IKEA effect and participant relationships on empathy toward agents

As AI, robotics, and anthropomorphic agents continue to be integrated into society, understanding the mechanisms that promote empathy toward these agents is crucial for improving human-agent interactions. While previous studies on the IKEA effect have focused primarily on increasing the perceived value of artifacts, this study is the first to demonstrate that the IKEA effect can also promote empathy for artifacts. Notably, the effect was found to significantly enhance cognitive empathy, suggesting that assembling an agent may encourage individuals to adopt the agent’s perspective and imagine its experiences.

Although H2 and H3 were not supported, the results indicate that the IKEA effect promotes empathy toward the agent regardless of the participants’ relationship during the assembly process. This suggests that the degree of assembly work and the opportunity to interact with the agent were not significantly influenced by participant relationships.

Furthermore, this study revealed a significant difference between empathy for the agent and empathy for the co-participant. Empathy for the agent did not reach the same level as empathy for the co-participant, suggesting that while the media equation posits that people treat artifacts as social entities, differences in empathy judgments persist between agents and humans.

Interestingly, while human-directed empathy was initially higher than agent-directed empathy, the gap between the two narrowed after the task—particularly for cognitive empathy. This suggests that empathy toward agents can be improved through interaction and effort investment, consistent with the IKEA effect. In contrast, human-directed empathy remained relatively stable, indicating that baseline interpersonal empathy may be less influenced by short-term tasks.

This differential pattern implies that empathy for agents is more malleable and responsive to context, whereas empathy for humans may stem from deeper social norms or pre-existing expectations. Such asymmetry in empathy improvement underscores the importance of interaction design when aiming to foster human-agent relationships.

These findings highlight the potential of the IKEA effect in fostering empathy toward agents, which may contribute to improving human-agent relationships. In practical applications, integrating assembly-based interactions could help address concerns about the acceptance of high-performance AI systems in society. Additionally, users’ understanding of agents may be enhanced through the assembly process. For instance, incorporating assembled agents in educational settings could provide effective learning experiences while simultaneously promoting empathy toward the agents, fostering long-term engagement and acceptance.

Previous studies on empathy agents, such as those by Tsumura and Yamada [30,31], have examined factors including agent appearance, self-disclosure, task difficulty, task content, agent expressions, and task success or failure. When combined with the present study, these findings contribute to the design of more empathic agents that are more readily accepted by users.

### Methodological considerations in empathy measurement

This study also contributes methodologically by demonstrating how empathy scales traditionally designed for human-human contexts can be adapted for human-agent interaction. We employed a modified version of the Interpersonal Reactivity Index (IRI), changing referential terms to suit either a LEGO agent or a human co-participant. This adaptation has precedent in recent HAI studies [30,31], and our internal consistency metrics (Cronbach’s alpha = 0.723–0.890) support its psychometric reliability.

By maintaining the original structure of the IRI while adapting its referents, we preserved comparability across empathy objects and retained theoretical alignment with affective and cognitive empathy subdimensions. This approach enables direct within-participants comparison of empathy toward human and non-human entities—something not always feasible with newly improved or unvalidated agent-specific scales.

However, further research should explore the construct validity of these adaptations in broader contexts, possibly comparing multiple empathy measures side-by-side to assess convergent and discriminant validity.

### Limitations

One limitation of this study is the variability in assembly time among participants. Since the experiment was conducted in pairs, the assembly process could not be fully standardized. Participants who completed the task earlier may have had different interaction times with the agent, potentially affecting both their empathy toward the agent and their relationship with the co-participant. Future studies should investigate whether post-assembly interactions influence empathy toward the agent.

Also, a potential methodological limitation is the physical co-presence of participants in the individual condition. Although participants in this condition assembled separate LEGO agents independently and without any collaboration, they were seated in the same room and could see each other. This co-presence may have introduced subtle social influences, such as heightened social awareness or incidental empathy, potentially affecting ratings. However, the goal of this study was not to isolate collaboration per se, but rather to investigate how different types of participant relationships—cooperative versus parallel—modulate empathy toward both agents and co-participants. In this context, co-presence was not treated as a confound, but as a controlled part of the experimental environment to reflect naturalistic human-agent interaction settings (e.g., classrooms, workshops, or offices). Completely isolating participants would have removed the social situational frame that we aimed to preserve in both conditions, and would have limited the ecological validity of the empathy measurements. Future research may further disentangle the specific effects of co-presence by systematically varying levels of physical and social separation.

Additionally, to simplify the assembly process, small components of the LEGO agent were pre-assembled by the experimenters, and a video guide was provided. In practical applications, assembly tasks may be guided by written manuals rather than videos. Future research should explore whether the level of difficulty in the assembly task affects the degree to which the IKEA effect promotes empathy toward the agent.

Another consideration is the difficulty level of the task itself. While this study demonstrated that the IKEA effect influences empathy, it remains unclear whether an excessively difficult assembly process could hinder this effect. Future research should examine how task difficulty impacts empathy toward agents and whether simpler or more complex tasks yield different results.

And then, this study did not record the precise duration of the assembly task for each participant, nor did it assess the potential cognitive or emotional load associated with differences in assembly time. Although participants were provided a brief period (approximately five minutes) to verify that the LEGO agent functioned correctly, prolonged or rushed assembly may have affected empathy ratings due to fatigue or frustration. Future studies should consider recording and statistically controlling for assembly duration.

Additionally, we did not assess participants’ prior experience with LEGO robotics or their attitudes toward technology. While the assembly procedure was designed to be straightforward, individual differences in technological familiarity or anxiety may have influenced participants’ engagement or emotional responses. We suggest that future research incorporate these variables to clarify their potential influence.

Although our sample included adult participants across a wide age range (18–52 years), we did not analyze age-related effects. Future studies could examine whether psychological responses such as empathy and perceived ownership toward agents differ across age groups.

Finally, while this study focused on participant relationships, it did not consider social relationships beyond the experimental setting. In real-world scenarios, individuals working together in an assembly task may have pre-existing social relationships that influence their interactions. Future studies should examine how social relationships impact empathy toward agents in collaborative settings.

## Conclusion

To solve the problem of agents not being accepted by humans, we hope that by encouraging humans to empathize with them, agents will be used more in human society in the future. This study is an example of how to promote empathy between humans and agents. The experiment was conducted with a three-factor mixed design, with the between-participants factor measuring the relationship between the participants and the within-participants factor measuring the change in empathy toward the target before and after the IKEA effect and with the empathy object. The results showed that there was no main effect for the participants’ relationality factor and that after the IKEA effect, more empathy was promoted toward the agent by a statistically significant margin. In addition, the participants’ relationship factor did not show an effect on empathy toward the agent, and the degree of assembly work did not affect empathy. These results supported one of our hypotheses. This study is an important example of how human empathy works with artifacts. Future work will investigate whether the IKEA effect also promotes empathy for agents in online environments and will focus on the impact of interactions after the IKEA effect.

## Supporting information

S1 File Complete data set. (XLSX)

S1 Video Initial demonstration video. (MP4)
